# Levels of Susceptibility to Exercise Dependence and Mood States in Street Runners

**DOI:** 10.3390/sports14080335

**Published:** 2026-08-04

**Authors:** Anderson Ricardo Malmonge Barbosa Luciano, Flávia Marchesi Ciniciato, Danilo Malmonge Barbosa Luciano, Ercizio Lucas Biazus, Vinicius Barroso Hirota, Elias De França, Marcelo Rodrigues da Cunha, Carlos Eduardo Lopes Verardi

**Affiliations:** 1 Graduate Program in Developmental and Learning Psychology, Faculty of Sciences, São Paulo State University “Júlio de Mesquita Filho” (UNESP), Bauru 17033-360, SP, Brazil; ercizio.lucas@unesp.br; 2 Department of Physical Education, Faculty of Sciences, São Paulo State University “Júlio de Mesquita Filho” (UNESP), Bauru 17033-360, SP, Brazil; flavia.ciniciato@unesp.br; 3 Department of Health Sciences, Faculdades Integradas de Bauru (FIB), Bauru 17056-120, SP, Brazil; malmonge97@gmail.com; 4 Physical Education Program, Faculty of Technology of Sports (FATEC), Centro Paula Souza (CPS), São Paulo 02180-021, SP, Brazil; vbhirota@gmail.com; 5 São Paulo Futebol Clube (SPFC), São Paulo 05653-070, SP, Brazil; elias.de.f@hotmail.com; 6 Postgraduate Program in Health Sciences, Faculty of Medicine of Jundiaí (FMJ), Jundiaí 13202-550, SP, Brazil; marcelocunha@g.fmj.br

**Keywords:** street running, exercise dependence, mood states

## Abstract

The regular practice of physical exercise is often associated with promoting benefits for practitioners. In recent decades, street running has become popular worldwide, with most participants being recreational runners who engage in the activity primarily to promote health, well-being, leisure, and quality of life, although some also participate with competitive goals. Among the psychological aspects related to exercise, studies have shown that some individuals may become susceptible to “exercise dependence,” a condition in which exercise takes a central role in a person’s life. As a result, individuals may exhibit characteristics such as excessive training, injuries; weight control issues, withdrawal symptoms when unable to exercise and even problems in social relationships when exercise is exclusively prioritized. Background/Objectives: The aim of this study was to investigate and analyze levels of susceptibility to running dependence and its relationship with exercise volume and mood states (tension, depression, anger, vigor, fatigue, and mental confusion). Methods: This was a quantitative cross-sectional study involving 761 regular Brazilian runners (59.8% male and 40.2% female) who consented to participate and answered the Sociodemographic Questionnaire, the Escala de Dependência de Corrida (EDC), a Brazilian Portuguese adaptation of the Negative Addiction Scale (NAS), and the Brunel Mood Scale (BRUMS). The analysis plan was conducted through descriptive statistics (mean, standard deviation, median, 1st and 3rd quartiles, interquartile range, and 95% confidence intervals) and inferential statistics (Spearman’s Correlation and Kruskal–Wallis Test). In all tests, the significance level adopted was *p* < 0.05. Results: The sample was divided into three groups of susceptibility to exercise dependence: low (37.6%), moderate (36.4%), and high (26%). Scores indicative of greater susceptibility to exercise dependence showed positive correlations with running practice volume (years of experience, weekly training frequency, weekly training duration, and weekly mileage), as well as with vigor. Although a statistically significant correlation was also observed for tension, its magnitude was trivial (rs = 0.080). Comparisons using the Kruskal–Wallis test showed that higher susceptibility levels were associated with greater running volumes and higher raw vigor scores, whereas no significant group differences were observed for the remaining mood states. Conclusions: Higher susceptibility to exercise dependence was associated with greater running volume and higher raw vigor scores. No consistent associations were observed with negative mood states, except for a trivial correlation with tension. These findings suggest that greater susceptibility to exercise dependence among runners was primarily associated with higher raw vigor scores rather than with broader alterations in mood states.

## 1. Introduction

It is well established in the academic literature that regular physical exercise promotes various health benefits for practitioners [1,2]. Among many practices, street running has recently gained prominence as one of the most practiced modalities in several countries. In recent decades, the international popularization of street running is reflected by the significant increase in the number of participants and the organization of events. In general, runners can be amateurs or professionals, and the motivations for this practice are diverse, involving various factors such as the pursuit of well-being, quality of life, sociability, body aesthetics, competitiveness, professional goals, and more [3,4]. Along with the increase in running practice, there has also been a growing number of scientific studies investigating the physical and psychological effects resulting from this physical exercise [5,6,7,8].

Despite the numerous benefits of physical exercise, studies have shown that for some individuals, the behavior of exercising can become compulsive and even harmful to their health. This phenomenon is referred to, among other terms, as “exercise dependence.” Although exercise dependence is not a widely discussed topic, its consequences can be highly destructive to individuals experiencing this condition, making it a subject of significant relevance for scientific studies [9,10,11].

Among the various practices, aerobic exercises and sports that involve high intensity (e.g., running, CrossFit, and cycling) have been identified as activities in which practitioners may significantly increase the volume and intensity of their sessions and training routines. This is because the series of vigorous efforts involved in these activities promotes short- and/or medium-term improvements in physiological and psychological aspects. As a result, these factors can serve as motivational drivers for some individuals to continuously increase their practices, to the point of developing compulsive behaviors towards them [12,13]. While the benefits of exercise are widely recognized, extreme levels of practice can lead to addictive behaviors with potentially harmful consequences for a minority of individuals [14].

The associations made between negative psychological aspects and exercise are found in investigations related to exercise dependence, which indicate that individuals with behavioral characteristics of excessive and compulsive exercise may exhibit certain negative symptoms, such as decreased physical vigor, symptoms of anxiety, depression, and increased fatigue and irritability [15]. Specifically, mood changes are primarily associated with periods when, for some reason, these individuals are unable to exercise [9,10,11,16].

Although the inclusion of exercise dependence as a mental disorder classification in the Diagnostic and Statistical Manual of Mental Disorders has been suggested, this inclusion has not yet occurred [10,15,17,18]. Furthermore, despite the existence of various instruments for assessing exercise dependence, their criteria are not yet conclusive, requiring further experimental research, as well as consistent control groups, better control of participant biases, and improved operational procedures to better define the diagnosis of exercise dependence [14,15]. There is also a need for greater consistency in the development of terminology and assessments, as problems in these areas lead to screening instruments that may evaluate susceptibility, presence, and intensity of symptoms and/or risks, but not provide a precise diagnosis of exercise dependence [18].

Based on this information, although the literature reports negative psychological consequences among individuals presenting characteristics associated with exercise dependence, these effects have mainly been described during periods of exercise deprivation rather than while regular exercise is maintained [11]. However, to date, few studies have explored the relationship between exercise dependence and mood states in runners under regular training conditions. Thus, investigations on this topic are relevant because they contribute to expanding knowledge about the association between susceptibility to exercise dependence and mood states in runners who have not been deprived of exercise. Therefore, based on the aspects discussed in this introduction, the aim of this study was to investigate the relationship between susceptibility to exercise dependence and the mood states of regular street runners who had not been deprived of their practices. The hypotheses considered were that practitioners with higher indicators of exercise dependence would exhibit greater running volumes and higher vigor scores, without marked increases in negative mood states, given that they had not been deprived of their regular running practices.

## 2. Materials and Methods

### 2.1. Participants

This study involved 761 regular street runners with a mean age of 39.00 ± 12.57 years, of both sexes, with 40.2% (306) being female and 59.8% (455) male.

### 2.2. Procedures

Initially, the research project was submitted for review by the Research Ethics Committee of São Paulo State University “Júlio de Mesquita Filho” (UNESP Bauru/São Paulo/Brazil) and approved according to the Process CAAE: 99303818.0.0000.5398, in compliance with the Guidelines and Regulatory Standards for Research Involving Human Beings of the National Health Council, Resolution 466/2012 [19].

**Figure 1:**
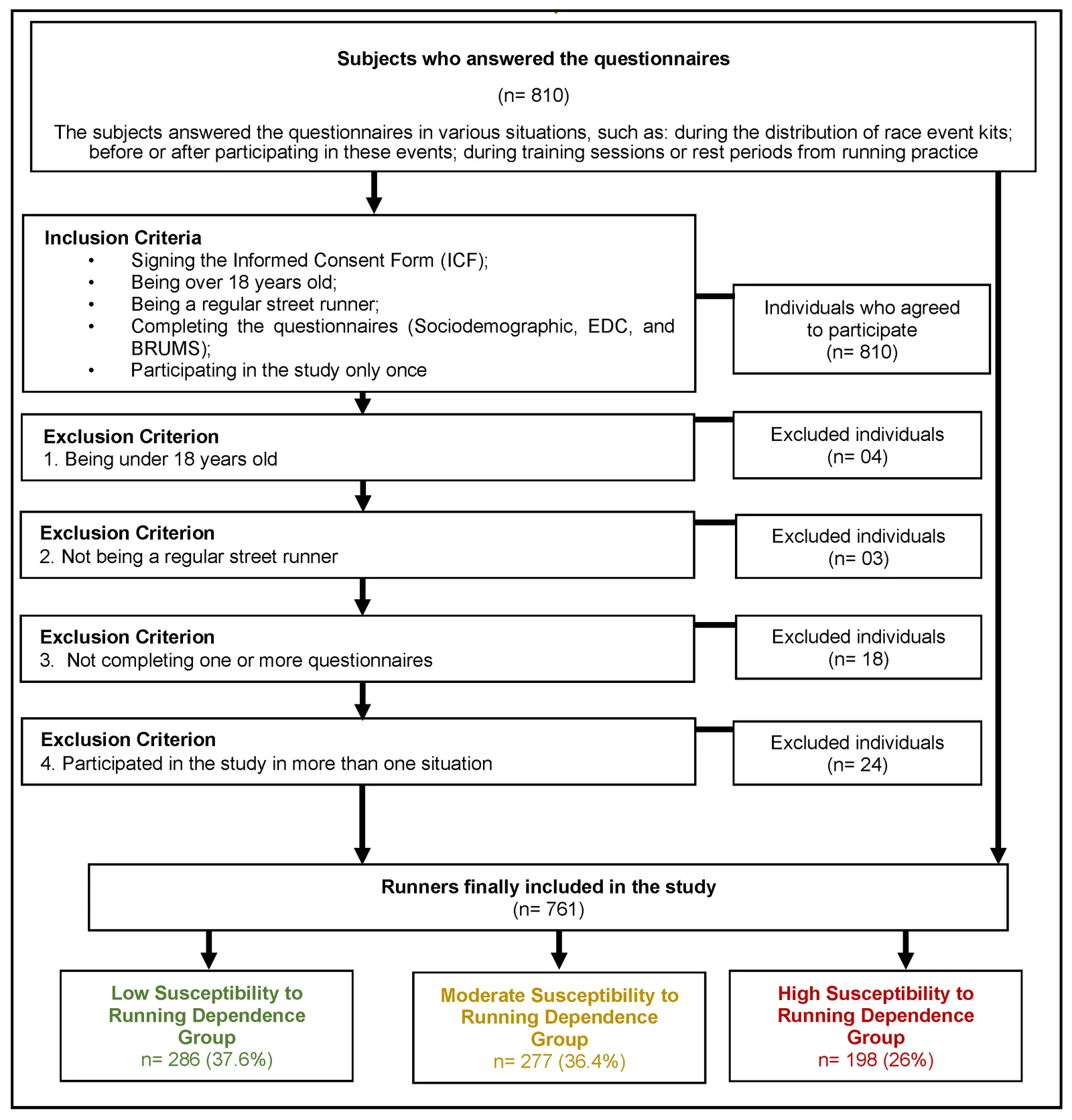
Flow diagram of participant inclusion and exclusion developed by the authors according to the STROBE guidelines. Abbreviations: ICF (Informed Consent Form), EDC (Escala de Dependência de Corrida, Brazilian Portuguese adaptation of the Negative Addiction Scale—NAS), and BRUMS (Brunel Mood Scale). Note: Figure 1 was originally developed by the authors. Its organization follows the general recommendations of the STROBE (Strengthening the Reporting of Observational Studies in Epidemiology) guidelines for reporting participant flow in observational studies and does not reproduce copyrighted material. See Malta et al. [20].

Before participating in the research, all volunteers were informed about the study’s objectives, as well as the procedures to be carried out and the preservation of their identities in relation to the dissemination of the research. Those who agreed to participate, before answering the questionnaires, read and signed an Informed Consent Form (ICF). The criteria adopted for the inclusion and exclusion of individuals are presented in the diagram below (Figure 1), organized in accordance with the STROBE guidelines [20].

### 2.3. Instruments

Sociodemographic Questionnaire: This instrument was used to collect sociodemographic data from the participants, including age, gender, weight, height, education level, profession, as well as characteristics related to running practice, such as duration of practice (years/months), participation in races, and training routines (days per week, time per session, weekly mileage, professional guidance, and the practice of other exercises/sports).

Escala de Dependência de Corrida (EDC; Running Dependence Scale): Originally developed by Hailey and Bailey [21], the Negative Addiction Scale (NAS) was translated and adapted for Brazilian runners by Rosa, Mello, and Souza-Formigoni [22]. The scale quantifies scores indicative of running dependence based on negative psychological aspects related to this physical exercise/sport. Through its application, a score of up to 14 points can be obtained, with higher scores indicating greater behavioral characteristics related to exercise dependence. In the present study, EDC scores were classified into levels of exercise dependence susceptibility (low (37.6%), moderate (36.4%), and high (26%)) and not as a psychological diagnostic criterion for exercise dependence.

Brunel Mood Scale (BRUMS): Adapted from the Profile of Mood States instrument [23], BRUMS was developed to allow for the rapid assessment of mood states in adult and adolescent populations by Terry, Lane, and Fogarty [24]. It was later translated and validated into Portuguese by Rohlfs et al. [25]. In the present study, participants rated the scale according to the following response criterion: “How have you been feeling over the past week of training, including today?” [25]. BRUMS contains 24 mood descriptors representing six subjective and transient mood dimensions: tension-anxiety (T), depression-dejection (D), anger-hostility (A), vigor-activity (V), fatigue-inertia (F), and confusion-bewilderment (C). Tension, depression, anger, fatigue, and confusion are considered negative mood dimensions, whereas vigor is considered a positive mood dimension. In the traditional interpretation of the Iceberg Profile, vigor is positioned above the normative mean, and the negative mood dimensions are positioned below it. This interpretation requires the use of normative standardized scores, commonly expressed as T-scores with a mean of 50 and a standard deviation of 10 [26]. In the present study, the BRUMS dimensions were analyzed using raw scores; therefore, no formal classification of the Iceberg Profile or Inverted Iceberg Profile was performed.

### 2.4. Statistical Analysis

To verify the normality of the data, the Kolmogorov–Smirnov Test was performed, with statistical significance set at *p* > 0.05. The data did not meet the normality assumptions (*p* < 0.05), and thus are considered non-parametric. A K-means cluster analysis was performed using the total Running Dependence Scale score to group participants with similar levels of susceptibility to exercise dependence. A three-cluster solution was specified a priori because it provided a conceptually meaningful classification into low, moderate, and high susceptibility, an approach commonly adopted in studies that classify participants according to levels of risk or susceptibility. Following the iterative clustering procedure, participants were allocated according to their proximity to the final cluster centers, resulting in the following score ranges: low susceptibility (0–3), moderate susceptibility (4–6), and high susceptibility (7–12). The solution converged after the ninth iteration.

Variables related to running practice volume and mood states were presented in descriptive statistics tables. BRUMS subscale scores were analyzed as raw scores and were not converted into normative T-scores [26]. Accordingly, the mood analyses focused on the associations between the individual BRUMS dimensions and susceptibility to exercise dependence, as well as on between-group comparisons, rather than on the formal classification of standardized mood profiles. Descriptive statistics included mean (x), standard deviation (sd), median (Md), interquartile range (IQR), and 95% confidence interval (CI 95%). For Inferential Statistics, Spearman’s Rank Correlation Coefficient (rs) was used to analyze correlations between running scores indicative of susceptibility to exercise dependence and aspects of running practice volume and the six mood states. The strength of the correlation coefficient was qualitatively assessed according to [27] as follows: null (0); weak (0.01 to 0.30); moderate (0.31 to 0.60); strong (0.61 to 0.90); very strong (0.91 to 0.99); and perfect (1). The Non-Parametric Kruskal–Wallis Test was used to compare the quantitative variables of participants’ characteristics and mood states among the three groups of susceptibility to running dependence. Statistical analysis was performed using IBM^®^ SPSS^®^ Statistics Version 25 software, and a significance level of *p* < 0.05 was adopted for all statistical tests.

Additionally, effect sizes for the Kruskal–Wallis comparisons were estimated using eta squared based on the Kruskal–Wallis H statistic (η^2^H), calculated as (H − k + 1)/(n − k), where H is the Kruskal–Wallis statistic, k is the number of groups, and n is the total sample size. Effect sizes were interpreted as negligible (<0.01), small (0.01 to <0.06), moderate (0.06 to <0.14), and large (≥0.14).

## 3. Results

According to the participants’ self-reports, Table 1 shows the quantitative variables related to the characteristics of running practice volume among the participants.

Table 2 presents the descriptive data from the EDC and BRUMS instruments. Regarding mood states, vigor showed the highest raw scores among the BRUMS dimensions (Md = 12.00; mean = 11.30; CI 95% = 11.10–11.51), compared with tension, depression, anger, fatigue, and mental confusion. Because these values represent raw scores rather than normative T-scores, they are reported descriptively and are not interpreted as evidence of a formal Iceberg Profile [26].

Spearman’s rank correlation coefficients were calculated to analyze the associations between running dependence scores, running practice volume variables, and the six mood-state dimensions (Table 3).

Table 3 shows that there was a “weak” positive correlation that was statistically significant between the EDC scores and years of practice (rs = 0.137; *p* < 0.001), as well as “moderate” positive correlations that were statistically significant between dependence scores and the number of days of running practice per week (rs = 0.400; *p* < 0.001), weekly practice time (rs = 0.395; *p* < 0.001), and weekly mileage (rs = 0.346; *p* < 0.001). Regarding mood states, a weak positive correlation was observed between dependence scores and vigor (rs = 0.296; *p* < 0.001). Although the correlation with tension reached statistical significance (rs = 0.080; *p* = 0.028), its magnitude was trivial. Higher volumes of running practice were positively associated with higher levels of susceptibility to exercise dependence.

The three-cluster solution converged after nine iterations of the K-means algorithm. The final cluster centers represented progressively higher levels of susceptibility to exercise dependence, allowing participants to be classified into low-, moderate-, and high-susceptibility groups, which were subsequently used in the comparative analyses.

A Cluster Analysis (K-means clustering) was conducted to group subjects with similar responses regarding the EDC. Based on this analysis, three groups of susceptibility to running dependence were defined: low (scores between 0 and 3), moderate (scores between 4 and 6), and high (scores between 7 and 12). The comparisons of the analyses of running practice volumes are presented in Table 4.

In relation to the time spent running (in years), the comparisons indicated significant differences between the groups, with the high susceptibility group showing greater practice time (Md: 4.00; IC95%: 5.73–7.94) compared to the low (Md: 3.04; CI 95%: 4.00–5.24) and moderate (Md: 3.50; CI 95%: 4.50–5.91) groups.

The number of days per week of running practice also showed statistically significant differences in comparisons between each pair of groups (*p* = 0.000), with higher values observed in groups with greater levels of susceptibility to dependence. The same trend was seen for time spent practicing and weekly mileage, as significant differences were found between the groups, with greater amounts of time and weekly mileage correlating with higher levels of susceptibility to running dependence. Effect size analysis indicated a small effect for running practice duration (η^2^H = 0.013) and moderate effects for days of running per week (η^2^H = 0.129), weekly training time (η^2^H = 0.131), and weekly mileage (η^2^H = 0.101), indicating that the most meaningful differences among susceptibility groups were related to training volume.

Table 5 presents the comparative analyses of the scores for the subscales of mood states among the three groups of susceptibility to running dependence. It is noted that the differences were not statistically significant among the groups (*p* > 0.05) for the negative factors of tension (*p* = 0.074), depression (*p* = 0.405), anger (*p* = 0.173), fatigue (*p* = 0.113), and confusion (*p* = 0.480). Statistically significant differences (*p* < 0.05) were identified only for the vigor factor (*p* = 0.000). Effect size analysis showed a moderate effect for vigor (η^2^H = 0.069), whereas all negative mood states presented negligible effect sizes (η^2^H ≤ 0.004), reinforcing that meaningful between-group differences were restricted to vigor.

## 4. Discussion

The objective of this study was to investigate the relationship between susceptibility to exercise dependence and mood states in regular runners. Although regular running practice is often associated with promoting well-being and quality of life due to its health benefits, some practitioners may develop harmful behaviors related to running [28].

Despite this phenomenon being addressed in the literature regarding behaviors characterized as “dependence,” which may share criteria similar to other addictions, there are still complexities concerning its diagnosis and, consequently, the definition of prevalence rates. This is due to the diversity of research types, terminology, procedures, instruments, exercise modalities, and sports investigated, as well as the varied profiles of samples assessed and the differing results found on the topic [13,18,29]. Therefore, this study did not consider the prevalence and/or diagnosis of exercise dependence, as this issue is complex and there are no well-defined criteria for diagnosing behaviors related to this type of dependence [14,18]. In this context, classifying participants into low-, moderate-, and high-susceptibility groups was intended for analytical rather than diagnostic purposes. The use of susceptibility levels enables comparisons between runners presenting different positions along the continuum of exercise dependence-related characteristics, an approach frequently adopted in the literature when investigating behavioral and psychological differences between participants with distinct levels of susceptibility. In the present study, these groups were derived through a K-means cluster analysis, allowing the classification to be based on the observed data distribution rather than on exclusively arbitrary cut-off points.

When evaluating the raw mood-state scores, vigor presented higher values than the negative mood dimensions. Tension and fatigue showed higher values than depression, anger, and confusion, but no significant between-group differences were identified for any of the negative mood dimensions. Although this distribution resembles the pattern traditionally associated with the Iceberg Profile, a formal Iceberg Profile cannot be established from raw scores alone because its interpretation requires appropriate normative T-scores [23,26]. Therefore, the present findings are more appropriately described as a mood pattern characterized by comparatively higher raw vigor scores and lower raw scores for the negative mood dimensions. This pattern is consistent with previous studies reporting comparatively favorable mood responses among individuals who regularly engage in physical exercise [30,31].

The tests used for inferential statistics identified positive and statistically significant correlations between the total scores of the EDC and the practice volume variables (years of practice, days per week, weekly duration, and weekly mileage), as well as with the mood state factors related to vigor and tension. When comparing groups, statistically significant differences were found in exercise practice volumes and vigor scores. The relationship between greater practice experience and higher risk levels for dependence has also been observed in other studies with gym-goers [32], bodybuilders [33], marathon runners [22], and runners [34].

The literature often discusses that individuals presenting higher indicators of susceptibility to exercise dependence tend to engage in higher training volumes in their practice routines [9,10,11,12,13,18]. In this context, the identification of positive and statistically significant relationships between indicators of running dependence and the frequency of practice (days per week), weekly duration, and weekly mileage confirms that practitioners with these indicators exercise more. It is important to highlight that, while greater amounts of physical exercise are associated with health benefits, the optimal frequencies, intensities, and durations of high-volume practices are still unclear [35]. In road running, research has noted that higher practice volumes are linked to increased occurrences of injuries [36].

Across all susceptibility groups, the raw BRUMS scores were characterized by comparatively higher vigor values and lower values for the negative mood dimensions. However, this distribution should not be classified as a formal Iceberg Profile because the scores were not converted into appropriate normative T-scores [26]. Similar findings were reported by Oliveira et al. [37] and Anderson et al. [38], who also observed no substantial mood disturbances among runners presenting indicators associated with exercise dependence while maintaining their regular training routines. These studies, like the present investigation, evaluated participants who had not been deprived of exercise, which may help explain the predominance of comparatively favorable mood responses.

Considering that the present sample consisted predominantly of recreational street runners who regularly practiced running for health, well-being, and quality-of-life purposes, the higher vigor scores observed may also reflect engagement with regular training and the positive psychological effects commonly associated with habitual physical exercise, rather than indicating the absence of potentially problematic exercise behaviors.

The fact that the runners assessed in this study were not deprived of their usual practices and presented comparatively higher raw vigor scores than scores for the negative mood dimensions highlights the importance of considering mood responses under habitual training conditions. Previous literature suggests that positive affective responses may contribute to motivation, adherence, and the maintenance of regular exercise [39].

It is important to emphasize, however, that higher raw vigor scores should not be interpreted as evidence that runners with greater susceptibility to exercise dependence are free from potentially problematic exercise behaviors. In recreational runners, higher vigor may simply reflect greater engagement with training, better physical conditioning, enjoyment of running, or the acute psychological benefits associated with regular exercise. At the same time, higher training volumes may still increase the likelihood of adverse outcomes, such as injuries, compulsive exercise patterns, or withdrawal symptoms during periods in which regular training cannot be maintained. Therefore, raw vigor scores should be interpreted as representing only one dimension of the psychological profile of these runners rather than as an indicator that susceptibility to exercise dependence is psychologically harmless.

Overall, participants showed a raw-score pattern characterized by comparatively higher vigor values and lower values for the negative mood dimensions. This distribution should not be interpreted as evidence of a formal Iceberg Profile because the analyses were not based on appropriate normative T-scores [26]. Although tension showed a statistically significant correlation with susceptibility scores, its magnitude was trivial (rs = 0.080), and no significant differences in tension were observed between the susceptibility groups. Therefore, this finding should be interpreted cautiously and does not provide strong evidence of meaningful mood alterations associated with greater susceptibility to exercise dependence. Although previous literature has discussed possible associations between compulsive exercise and anxiety-related characteristics [40], the present findings provide only limited support for this interpretation. Regarding fatigue, the slightly higher values observed in groups with greater susceptibility may be related to their higher weekly exercise volumes, potentially contributing to a greater perception of fatigue [6].

The findings of this study relate to those of Glasser [41], who identified that habitual runners, even with high weekly volumes of running, exhibit a set of positive psychological aspects that positively influence overall well-being, which he attributes to a condition of “positive addiction”.

This study has limitations regarding the instrument used to assess indicators of susceptibility to running dependence, the EDC. Hausenblas and Downs [15] argue that this instrument has deficiencies, as responses to some questions may be confused with participants’ levels of negative emotion rather than their participants’ susceptibility to exercise dependence. Furthermore, the authors note that the validity and reliability of this instrument are unknown. In addition to the limitations of the instrument used, it is important to consider how the “addiction” to running is perceived within the community of practitioners and how this perception may influence responses to the questionnaire. Many practitioners may not view their “addiction” to running as detrimental, which can lead some subjects to equate a strong sense of “commitment” and “involvement” with the activity as synonymous with “addiction,” which would not be linguistically correct due to the different meanings of these expressions [11,42].

Another limitation of this study is its cross-sectional design, which does not allow causal relationships to be established between exercise dependence indicators, running practice volume, and mood states. Therefore, the findings should be interpreted as associations rather than cause-and-effect relationships.

An additional aspect that should be considered is the context in which the BRUMS questionnaire was administered. Participants completed the questionnaires under different circumstances, including during race-kit distribution, before or after running events, during training sessions, and during periods of rest. Because mood states are transient and may be influenced by factors such as anticipation, acute fatigue, or recent exercise, these different assessment contexts may have affected BRUMS scores. Although this reflects the ecological conditions under which recreational runners are commonly assessed, future studies should seek to standardize the timing of mood assessment or statistically control for assessment context whenever possible [26].

A further limitation concerns the interpretation of the BRUMS scores. The present analyses were conducted using raw subscale scores, without conversion to normative T-scores. Because the formal characterization of the Iceberg Profile requires comparison with appropriate normative values, the distribution observed in the present study should not be interpreted as evidence of a standardized Iceberg Profile [26]. Moreover, the athletic norms proposed by Terry and Lane were developed for the original POMS using a “right now” response set and should not be applied automatically to the BRUMS scores collected over the previous week. Future studies should consider using normative data that are appropriate to the instrument, population, language, and response period when the objective is to characterize standardized mood profiles.

Although the participants were regular street runners and the sample predominantly represented recreational runners, additional characteristics such as competitive level, current training phase, injury status, preparation for a specific event, and coaching status were not examined in the present analyses. These factors may influence both susceptibility to exercise dependence scores and mood states and should therefore be considered in future investigations to provide a more comprehensive understanding of these relationships.

Although formal adjustments for multiple comparisons were not applied, the analyses were predefined according to the study objectives and interpreted considering both statistical significance and effect size magnitude. Future studies may consider applying formal procedures to control the family-wise error rate.

## 5. Conclusions

Higher susceptibility to exercise dependence was associated with greater running practice volume, including years of experience, weekly training frequency, weekly training duration, and weekly mileage. Participants with higher susceptibility also presented higher raw vigor scores. Although a statistically significant correlation with tension was observed, its magnitude was trivial, and no consistent associations were identified with the remaining negative mood states.

These findings suggest that, under regular training conditions, greater susceptibility to exercise dependence was primarily associated with higher raw vigor scores rather than with broader alterations in mood states. Because the BRUMS scores were not converted into appropriate normative T-scores, the observed distribution should not be interpreted as evidence of a formal Iceberg Profile. Nevertheless, because greater susceptibility was also associated with higher training volumes, runners should be encouraged to maintain appropriate training loads in order to reduce the risk of injuries and other adverse consequences associated with excessive exercise.

Although a statistically significant association between exercise dependence indicators and tension scores was observed, its magnitude was trivial and should therefore be interpreted with caution. Future longitudinal studies are warranted to clarify whether this relationship has clinical relevance under different training conditions.

The research field to which this study belongs, namely exercise dependence, is still under development. Therefore, future studies should seek to improve the terminology and assessment instruments related to indicators of exercise dependence.

## Figures and Tables

**Table 1:** Descriptive statistics related to running practice volume.

Variables	x ± sd	Md	Quartiles		IQR	CI 95%
			Q1	Q3		
Running practice duration (years)	5.41 ± 6.36	3.50	1.25	6.25	5.00	(4.96–5.86)
Days per week	3.20 ± 1.21	3.00	2.00	4.00	2.00	(3.12–3.29)
Time per week (minutes)	199.22 ± 148.19	180.00	120.00	240.00	120.00	(188.67–209.76)
Weekly mileage (km)	23.7 ± 17.81	20.00	12.00	30.00	18.00	(22.41–24.94)

Abbreviations: mean (x); standard deviation (sd); median (Md); 1st quartile (Q1); 3rd quartile (Q3); interquartile range (IQR); 95% confidence interval (CI 95%).

**Table 2:** Descriptive statistics of running dependence scores and mood states.

Variables	x ± sd	Md	Quartiles		IQR	CI 95%
			Q1	Q3		
**EDC**	4.63 ± 2.69	4.00	2.00	7.00	5.00	(4.43–4.82)
**BRUMS**						
Tension	2.57 ± 2.74	2.00	0.00	4.00	4.00	(2.37–2.76)
Depression	0.54 ± 1.26	0.00	0.00	0.00	0.00	(0.45–0.63)
Anger	0.67 ± 1.54	0.00	0.00	0.00	0.00	(0.56–0.78)
Vigor	11.30 ± 2.88	12.00	9.00	13.00	4.00	(11.10–11.51)
Fatigue	2.85 ± 2.84	2.00	0.00	4.00	4.00	(2.64–3.05)
Mental confusion	0.84 ± 1.65	0.00	0.00	1.00	1.00	(0.73–0.96)

Abbreviations: mean (x); standard deviation (sd); median (Md); 1st quartile (Q1); 3rd quartile (Q3); interquartile range (IQR); 95% confidence interval (CI 95%); Escala de Dependência de Corrida (EDC); Brunel Mood Scale (BRUMS).

**Table 3:** Spearman’s Rank Correlation between dependence scores, running practice volume variables, and mood states.

Variables		Running Practice Volume				Mood States					
		Years of Practice	Days per Week	Weekly Time	Weekly Mileage	T	D	A	V	F	C
Running Dependence	*rs*	0.137 **	0.400 **	0.395 **	0.346 **	0.080 *	−0.014	0.012	0.296 **	0.031	−0.005
	*p*	0.000	0.000	0.000	0.000	0.028	0.703	0.751	0.000	0.401	0.895

Abbreviations: Tension (T); Depression (D); Anger (A); Vigor (V); Fatigue (F); Mental Confusion (C); Spearman’s rho coefficient (*rs*); statistical significance (*p*). Note: *. Correlation is significant at the 0.05 level (2-tailed). **. Correlation is significant at the 0.01 level (2-tailed).

**Table 4:** Comparison of quantitative variables of running practice volume among the risk groups (low, moderate, and high) for dependence, according to the Kruskal–Wallis Test.

	x ± sd	Md	Quartiles		IQR	CI 95%	H	df	*p*	
			Q1	Q3						η^2^H
**Running Practice Time (years)**										
Low	4.62 ± 5.29	3.04	1	5.95	4.95	(4.00–5.24)	12.094	2	0.002 *	
Moderate	5.21 ± 6	3.5	1.17	6	4.83	(4.50–5.91)				0.013
High	6.83 ± 7.9	4	2	8	6	(5.73–7.94)				
**Days per week**										
Low	2.71 ± 1.02	3	2	3	1	(2.59–2.83)	99.591	2	0.000 *	
Moderate	3.29 ± 1.17	3	3	4	1	(3.15–3.43)				0.129
High	3.8 ± 1.24	3	3	4	1	(3.62–3.97)				
**Time per week (minutes**)										
Low	148.99 ± 103.71	120	80	180	100	(136.92–161.06)	101.22	2	0.000 *	
Moderate	213.5 ± 154.34	180	120	240	120	(195.25–231.76)				0.131
High	251.78 ± 170.47	205	160	300	140	(227.89–275.67)				
**Weekly mileage (km)**										
Low	18.14 ± 13.4	15	10	20.75	10.75	(16.57–19.69)	78.054	2	0.000 *	
Moderate	25 ± 19.03	20	14	30	16	(22.72–27.23)				0.101
High	29.9 ± 19.24	25	15	38	23	(27.18–32.57)				

Abbreviations: mean (x); standard deviation (sd); median (Md); 1st quartile (Q1); 3rd quartile (Q3); interquartile range (IQR); 95% confidence interval (CI 95%); Kruskal–Wallis H (H); degrees of freedom (df); statistical probability (*p*); eta squared based on the Kruskal–Wallis H statistic (η^2^H). Note: * Statistically significant difference (*p* < 0.05); Effect sizes were interpreted as negligible (<0.01), small (0.01 to <0.06), moderate (0.06 to <0.14), and large (≥0.14).

**Table 5:** Comparison of mood scores among the three groups of risk levels for running dependency, based on the Kruskal–Wallis Test.

Variables	Running Dependence																					
	Low Risk n = 286 (37.6%)						Moderate Risk n = 277 (36.4%)						High Risk n = 198 (26%)									
	x ± sd	Md	Quartiles		IQR	CI 95%	x ± sd	Md	Quartiles		IQR	CI 95%	x ± sd	Md	Quartiles		IQR	CI 95%	H	df	*p*	η^2^H
			Q1	Q3					Q1	Q3					Q1	Q3						
**BRUMS**																						
Tension	2.23 ± 2.44	1.5	0	4	4	(1.95–2.52)	2.65 ± 2.76	2	0	4	4	(2.33–2.98)	2.92 ± 3.06	2	0	4	4	(2.49–3.35)	5.216	2	0.074	0.004
Depression	0.5 ± 1.22	0	0	0	0	(0.36–0.64)	0.61 ± 1.34	0	0	1	1	(0.45–0.77)	0.51 ± 1.18	0	0	0	0	(0.34–0.67)	1.808	2	0.405	0.000
Anger	0.59 ± 1.51	0	0	0	0	(0.41–0.76)	0.78 ± 1.61	0	0	1	1	(0.59–0.97)	0.65 ± 1.49	0	0	0	0	(0.44–0.86)	3.507	2	0.173	0.002
Vigor	10.45 ± 2.74	11	9	12	3	(10.14–10.77)	11.48 ± 2.95	12	10	14	4	(11.13–11.83)	12.28 ± 2.63	12	11	14	3	(11.91–12.65)	53.993	2	0.000 *	0.069
Fatigue	2.48 ± 2.46	2	0	4	4	(2.20–2.77)	3.1 ± 2.96	3	0	5	5	(2.75–3.45)	3.02 ± 3.14	2	0	4.75	4.75	(2.58–3.46)	4.369	2	0.113	0.003
Mental Confusion	0.8 ± 1.54	0	0	1	1	(0.62–0.98)	0.94 ± 1.74	0	0	1	1	(0.73–1.14)	0.78 ± 1.69	0	0	1	1	(0.54–1.01)	1.469	2	0.480	0.000

Abbreviations: mean (x); standard deviation (sd); median (Md); 1st quartile (Q1); 3rd quartile (Q3); interquartile range (IQR); 95% confidence intervals (CI 95%); Kruskal–Wallis H (H); degrees of freedom (df); statistical probability (*p*); BRUMS (Brunel Mood Scale); eta squared based on the Kruskal–Wallis H statistic (η^2^H), representing the effect size of between-group differences. Note: * Statistically significant difference (*p* < 0.05); Effect sizes were interpreted as negligible (<0.01), small (0.01 to <0.06), moderate (0.06 to <0.14), and large (≥0.14).

## Data Availability

The data presented in this study are available from the corresponding author upon reasonable request. The data are not publicly available due to privacy and ethical restrictions related to the participants involved in the study.

## Abbreviations

The following abbreviations are used in this manuscript:

BRUMS	Brunel Mood Scale
CAAE	Certificado de Apresentação para Apreciação Ética (Certificate of Presentation for Ethical Consideration)
CI 95%	95% Confidence Interval
df	Degrees of Freedom
EDC	Escala de Dependência de Corrida, Brazilian Portuguese adaptation of the Negative Addiction Scale (NAS)
H	Kruskal–Wallis H Statistic
ICF	Informed Consent Form
IQR	Interquartile Range
Md	Median
NAS	Negative Addiction Scale
POMS	Profile of Mood States
Q1	First Quartile
Q3	Third Quartile
rs	Spearman’s Rank Correlation Coefficient
SD	Standard Deviation
SPSS	Statistical Package for the Social Sciences
STROBE	Strengthening the Reporting of Observational Studies in Epidemiology
UNESP	Universidade Estadual Paulista “Júlio de Mesquita Filho” (São Paulo State University “Júlio de Mesquita Filho”)
T-score	Standardized T-score
η^2^H	Eta squared based on the Kruskal–Wallis H statistic (effect size)
